# A retrospective analysis on the correlation between hip pain, physical examination findings, and alpha angle on MR images

**DOI:** 10.1186/s13018-016-0476-9

**Published:** 2016-11-15

**Authors:** Olcay Guler, Mehmet Isyar, Dilek Karataş, Tugrul Ormeci, Halis Cerci, Mahir Mahirogulları

**Affiliations:** 1 Orthopedics and Traumatology Department, Medical Faculty, Medipol University, Atatürk Bulvarı No: 27 Unkapanı, 34083 Fatih, Istanbul Turkey; 2 Department of Radiology, Nisa Hospital, Istanbul, Turkey; 3 Department of Radiology, Medical Faculty, Medipol University, Istanbul, Turkey; 4 Department of Orthopedics and Traumatology, Nisa Hospital, Istanbul, Turkey

**Keywords:** Femoroacetabular impingement, Alpha angle, Internal rotation, Hip, Pain

## Abstract

**Background:**

We aimed to search whether alpha angle, a radiological clue used in the diagnosis of femoroacetabular impingement, is correlated with the presence of hip pain, internal rotation angle, and impingement test results on hip impingement patients (CAM type).

**Methods:**

Medical records of 334 patients (156 women, 178 men) with an average age of 33.8 ± 8.4 (range 20–50) years were retrospectively studied for the alpha angle of the hip measured on magnetic resonance images (MRI). Hip pain and internal rotation angles as well as results of impingement tests were reviewed.

**Results:**

Hip pain was reported more frequently on the right side (*n* = 35, 10.5%) compared to the left side (*n* = 22, 6.6%) (*p* = 0.047). No difference was observed between the right and left sides regarding alpha angles (*p* = 0.145), internal rotation angles (*p* = 0.637), or positivity of impingement test (*p* = 0.210). Internal rotation angles were significantly higher in cases without hip pain (*p* < 0.001) and in patients with negative impingement test result (*p* < 0.001). Internal rotation angle correlated positively with age and negatively with the alpha angle. Alpha angle was increased in cases that report pain, those with an internal rotation angle <20°, or cases with positive impingement test. The pain was more common, internal rotation angle was higher, and positivity for impingement was more frequent if the alpha angle was <55°. Patients with hip pain or positive impingement test or internal rotation angle <20° had increased alpha angles (*p* < 0.001).

**Conclusions:**

The pain, impingement test results, and internal rotation angle seem to be associated with alpha angle of the hip measured on MRI in hip impingement patients.

## Background

Femoroacetabular impingement (FAI) is a morphological disorder of the hip joint that shares a similar mechanical etiology with osteoarthritis [1]. A certain amount of idiopathic hip arthritis cases may be linked with FAI [1]. This pathology may occur due to bone abnormalities caused by overcoverage of the acetabulum (pincer type), asphericity of the femoral head and neck (cam type), or a combination of these conditions [2]. Femoroacetabular impingement may trigger cartilage destruction and give rise to osteoarthritis (OA) of the hip, which may present clinically as hip pain and restriction of movement [3].

The alpha angle is defined by Nötzli et al. to evaluate the asphericity of the head of the femur in magnetic resonance image (MRI) views [4]. It allows the assessment of the contour deformity of the femoral head-neck junction and may aid in setting the guidelines for treatment [5].

Timely diagnosis and appropriate treatment are crucial for the reduction of pain, improvement of function and prevention, or at least delay of OA. Early detection is especially important since the restoration of function may not be feasible after end-stage OA has occurred. Although early diagnosis is particularly important, misdiagnosis by clinicians unfamiliar with the disease is not uncommon. Therefore, integrated assessment of clinical and radiological findings is imperative for the identification of FAI. From this viewpoint, the definition of practical, reliable, and useful radiological tips and elucidation of the relationship between descriptive, clinical, and radiological variables will be critical to avoid a delay in diagnosis [1, 6].

In addition to radiological measures, clinical examination is crucial for screening asymptomatic abnormalities of FAI. The impingement test is highly sensitive to the induction of hip pain in symptomatic FAI patients. Symptomatic FAI patients frequently exhibit a limited range of motion involving flexion, abduction, adduction, and internal and external rotation [7]. Moreover, internal and external rotation and abduction are significantly correlated to alpha angle in symptomatic FAI patients [8]. However, whether these tests can be used on a population that is largely asymptomatic and only complains of hip pain for diagnostic and screening purposes is unclear.

The aim of the present study was to determine the factors affecting hip pain, internal rotation angle, alpha angle, and impingement test results and to investigate a correlation between alpha angle and these parameters in FAI patients (CAM type).

## Methods

### Study design

This retrospective study was implemented to study the relationship between impingement test, hip alpha angle, and hip pain for 3 months by using data derived from the medical files of 378 FAI patients (aged 20 to 50 years) admitted to the orthopedics and traumatology department of our tertiary care center and had hip MRI examination. Approval from the local Institutional Review Board was obtained before the study (Istanbul Medipol University Ethics Committee, date 25/06/2015, no 108400987-358).

Exclusion criteria were as follows: the presence of rheumatologic diseases (2 patients), arthrosis causing narrowing of the joint (3 patients), the presence of chondral injury or labrum tear on magnetic resonance images (MRI) (11 patients), pregnancy, history of previous hip surgery (5 patients) or hip disease during childhood (1 patient), lateral central edge angle was <20° on anteroposterior hip x-ray examination (6 patient), crossover sign, coxa profunda or protrusio acetabuli on anteroposterior pelvic x-ray examination (9 patient), retrovert acetabuli (4 patient), and femoral internal rotation angle >60° while on physical examination in supine position (3 patient). As a result, 44 patients were excluded and 334 patients were included to the study.

### Impingement test

The impingement test results were derived from the medical files of the patients. The test was performed in supine position by the same orthopedician. The hip joint was brought to passive flexion at 90°, together with adduction and internal rotation. A verbal report of pain during this maneuver was interpreted as a positive test result [9].

### Internal rotation angle

The degree of internal rotation was derived from the medical files of the patients. Physical examination was performed in supine position by the same orthopedician. The degree of internal rotation was measured using a goniometer while the hip was at 90° flexion. Abduction and adduction of the hip were limited due to pressure applied downward on the knee. The ankle was used to rotate the hip internally and externally [10].

### Magnetic resonance imaging

All imaging studies were conducted on a 1.5-Tesla MRI device (Sigma HDXT, General Electric, Chicago, IL, USA). Patients were in supine position while the hip joint was maintained in neutral position. Pulse sequence parameters of the turbo spin-echo sequence were as follows: repetition time (Tr), 637 ms; echo time (Te), 14 ms; field of view (FOV), 350 × 350 mm; matrix, 512 × 256; slice thickness, 3 mm; flip angle, 150°. In addition, we used a coronal T1-weighted sequence (Tr, 530 ms; Te, 14 ms; FOV, 400 × 400 mm; slice thickness, 5 mm; flip angle, 150°), axial oblique T1-weighted sequence (Tr, 530 ms; Te, 14 ms; FOV, 350 × 265 mm; slice thickness, 5 mm; flip angle, 150°) oriented along the axis of the femoral neck, and fat-suppressed T1-weighted fast low angle shot (FLASH) sequences (Tr, 795 ms; Te, 11 ms; FOV, 400 × 400 mm; slice thickness, 3 mm; flip angle, 60°).

### Measurement of alpha angles on MRI views

**Fig. 1:**
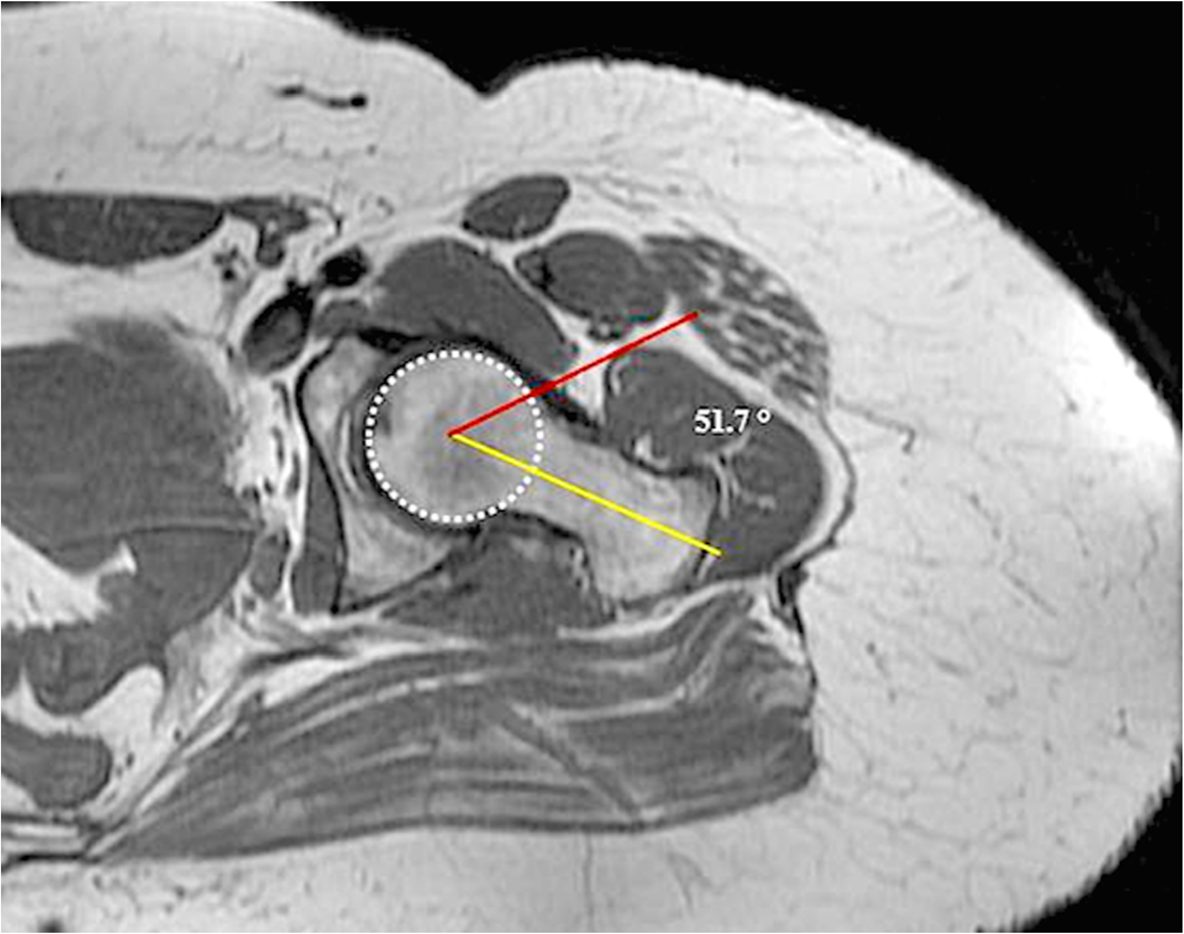
Measurement of alpha angle on MRI view of the left hip of a 24-year-old woman (the *yellow line* extends from the center of the femoral head to the midpoint of the femoral neck; the *red line* is extended from the center of the femoral head to the deviation of the femoral neck from the *circle* drawn around the femoral head)

Alpha angle was measured separately by two radiologists (DK, TO) who were blinded to patient data using the method described by Nötzli et al. [4] For this purpose, the Picture Archiving and Communication System (PACS, General Electric, Chicago, IL, USA) was used. Accordingly, the alpha angle of the hip was defined as the angle between two intersecting lines at the center of the femoral head. Using a best-fit circle digitized around the femoral head, the first line was extended from the center of the femoral head to the mid-point of the femoral neck. The second line was drawn from the center of the femoral head to the deviation of the femoral neck from the circle drawn around the femoral head (Fig. 1).

### Statistical analysis

A total of 334 cases (668 hips) were recruited. As the expected prevalence was reported to be 15%, this number allowed us to estimate the 95% confidence interval (CI) with a 5% margin of error and power of 90% [11]. Descriptive statistics (mean, standard deviation, median, minimum, and maximum) were used for continuous variables. Qualitative variables were compared with the chi-square test, while categorical dependent variables were studied using the McNemar test. Two independent groups that were not distributed normally were compared using the Mann-Whitney *U* test. Student’s *t* test was used to evaluate two independent groups with a normal distribution. The Wilcoxon Signed Rank test was used to compare two dependent variables that were not distributed normally. The level of significance was set at *p* < 0.05, and all analyses were implemented using the MedCalc Statistical Software program version 12.7.7 (MedCalc Software bvba, Ostend, Belgium; http://www.medcalc.org; 2013). Cronbach’s alpha, which indicates the average inter-correlation for the two radiologists who carried out measurements for the right and left hips, were 0.932 and 0.928, respectively.

## Results

An overview of the data is shown in Tables 1, 2, and 3. Our series consisted of 156 women (46.7%) and 178 men (53.3%), with an average age of 33.8 ± 8.4 years (range, 20 to 50). No difference was detected between women and men regarding age (*p* = 0.885). Average alpha angle was 53.1 ± 1.9 (range, 48.9 to 62.0). There was no significant difference between the right and left sides (*p* = 0.145).

**Table 1:** Alpha angle, hip pain, positive impingement test, and internal rotation angle in the right and left hips

Variable	Hip involvement		*p* value
	Right	Left	
Alpha angle	53.2 ± 2.0	53.1 ± 2.0	0.145
Hip pain (yes/no)	35/299	32/302	0.047
Positive impingement test	14/344	8/334	0.210
Internal rotation angle	31.7 ± 10.0	31.8 ± 9.4	0.637

**Table 2:** Average values for internal rotation angles with respect to the presence of hip pain and positive impingement test

Side	Hip pain		*p* value	Impingement test		*p* value
	(−)	(+)		(−)	(+)	
Right	33.6 ± 8.5	15.3 ± 5.3	<0.001	32.4 ± 9.6	16.8 ± 5.8	<0.001
Left	32.9 ± 8.6	16.8 ± 7.5	<0.001	32.2 ± 9.1	16.3 ± 6.4	<0.001

**Table 3:** Alpha angle, presence of hip pain, and positive impingement test in various subgroups

	Internal rotation angle		*p* value	Impingement test		*p* value	Hip pain		*p* value
	<20°	≥20°		(−)	(+)		(−)	(+)	
Alpha angle	56.7 ± 2.3	52.8 ± 1.6	<0.001	53.0 ± 1.8	56.1 ± 2.6	<0.001	52.7 ± 1.5	55.9 ± 2.2	<0.001
Hip pain (yes/no)	22/26	25/283	<0.001	31/315	16/19	<0.001	–	–	
Positive impingement test	10/6	9/299	<0.001	–	–		–	–	

Report of pain in the right hip (10.5%) was more frequent than in the left hip (6.6%) (*p* = 0.047), but results of the impingement test (*p* = 0.210) and internal rotation (*p* = 0.637) were similar. For both hips, the angle of internal rotation was significantly increased in the absence of hip pain and in patients with a negative impingement test (*p* < 0.001 for both).

In cases with an angle of internal rotation <20°, alpha angle and the likelihood of positivity of the impingement test were significantly increased (*p* < 0.001 for both). There was a moderate and negative correlation between internal rotation and alpha angle (*r* = −0.555; *p* < 0.001).

The pain was reported more frequently in patients with positive impingement test results (*p* < 0.001). Patients that reported pain were younger (*p* = 0.040) and had increased alpha angles (*p* < 0.001).

Cases with alpha angles ≥55° were younger (*p* = 0.005), suffered more frequently from pain (*p* < 0.001), and were more likely to have positive impingement test results (*p* < 0.001). The degree of internal rotation was higher in cases with alpha angles <55° (*p* < 0.001).

## Discussion

The purpose of the present study was to determine whether alpha angle was correlated with the presence of hip pain, internal rotation angle, and impingement test results in FAI patients (CAM type). Our results indicated that hip pain occurred more frequently on the right side, and internal rotation angles were significantly higher in cases that did not report hip pain and in those with a negative impingement test result. Internal rotation angle correlated positively with age and negatively with the alpha angle. Alpha angle was increased in cases that reported pain, those with an internal rotation angle <20°, or cases with a positive impingement test. The pain was more common, internal rotation angle was higher, and there were a greater number of positive impingements when the alpha angle was <55°. Patients with hip pain or a positive impingement test or an internal rotation angle <20° had significantly increased alpha angles. These results imply that alpha angle may be associated with clinical parameters and thus, may serve as a valuable marker for screening and diagnosing patients having complaints consistent with FAI.

Active and young people may suffer from groin pain due to internal rotation of the hip at 90° flexion. In such circumstances, the likelihood of anterior impingement of the femoral neck on the acetabular rim or labrum must be taken into account [12].

Osteoarthritis of the hip is a multifactorial disease linked to systemic and local risk factors such as degeneration due to slipped capital femoral epiphysis, developmental dysplasia of the hip, and Legg-Calvé-Perthes disease [13]. Nevertheless, these theories cannot fully explain cases with early OA. Some subtle structural alterations in the proximal femur or acetabulum have been associated with OA of the hip [11]. Since only a few data exist regarding the prevalence of radiographic changes in asymptomatic individuals, elucidation of the natural course of and its link with OA have not been possible to date [11].

Alpha angle was initially described by Notzli as an indicator of the loss of femoral head sphericity and, therefore, a marker of cam-type FAI [4]. A cut-off value was randomly determined between normal and abnormal angles; values >50° were considered abnormal [4]. The lack of uniformity in this criterion is reflected in our results, and similar to a report by Diesel, the cut-off value for alpha angle in our series was determined to be 55° [12]. In the literature, it has been reported that the cut-off value for a normal alpha angle can range from 42° to 68° [14].

Analysis of the relationship between hip deformities and the risk of OA development revealed that the non-spherical shape of the femoral head and enlargement of the femoral neck were linked with increased risk. However, these alterations may be a consequence of OA rather than a cause [15].

Our results were in accordance with the report by Kapron et al., suggesting that internal rotation measured in the supine position negatively correlates with alpha angle [2]. Pain may be elicited only in cases with underlying chondrolabral damage, and therefore, it must be remembered that the impingement test may be used to distinguish underlying chondrolabral damage [13].

Barton et al. suggested that radiographs might be sufficient for evaluation of the femoral head-neck junction [5]. In FAI, diagnostic imaging should be considered as a complementary measure to the clinical evaluation rather than being the sole tool for diagnosis [16]. The cam deformity may be diffuse along the femoral head-neck junction rather than being focal [17].

Magnetic resonance imaging is a specific imaging tool used to assess groin and hip pain. Since there is no standard test for such abnormalities, we hope that alpha angle may aid in the determination of the femoral head-neck relationship on MRI scans. Although there is much debate over the radiographic cut-off value for FAI diagnosis, we hope that our efforts will aid in establishing a standard screening protocol. From this point of view, the cut-off value of 55° for alpha angle and the negative correlation between internal rotation and alpha angle are important. Moreover, hip pain, impingement test positivity, and an internal rotation angle <20° were associated with increased alpha angle. A cautious and integrative assessment of physical examination findings and radiological data is mandatory to establish timely and correct diagnoses. Our findings imply that alpha angle is correlated with hip pain, and impingement test results and internal rotation and assessment of alpha angle in patients complaining of hip or groin pain may yield valuable data for both treatment and follow-up. Even though the accuracy of the alpha angle measurement on plain radiographs has been shown, MRI still stays as a powerful diagnostic measure that provides prognostic information and allows patient counseling [5].

Limitations of the present study include the lack of radiographic cut-off values and possible influences of genetic, ethnic, and environmental factors on the parameters under investigation. Reliability of the measurements with a goniometer is doubtful due to the personal variability of the anatomic landmarks. Moreover, measurements of internal rotation were not performed in prone and sitting positions. It must be noted that this study reflects the experience of a single institution, and the possibility of error and bias cannot be completely eliminated.

## Conclusions

The pain, impingement test results, and internal rotation angle seem to be associated with alpha angle of the hip measured on MRI in hip impingement patients.

## Abbrevıatıons

FAI: Femoroacetabular impingement
MRI: Magnetic resonance images
OA: Osteoarthritis
